# The Observation of Highly Ordered Domains in Membranes with Cholesterol

**DOI:** 10.1371/journal.pone.0066162

**Published:** 2013-06-18

**Authors:** Clare L. Armstrong, Drew Marquardt, Hannah Dies, Norbert Kučerka, Zahra Yamani, Thad A. Harroun, John Katsaras, An-Chang Shi, Maikel C. Rheinstädter

**Affiliations:** 1 Department of Physics and Astronomy, McMaster University, Hamilton, Ontario, Canada; 2 Department of Physics, Brock University, St. Catherines, Ontario, Canada; 3 Canadian Neutron Beam Centre, National Research, Council of Canada, Chalk River, Ontario, Canada; 4 Neutron Sciences Directorate, Oak Ridge National Laboratory, Oak Ridge, Tennessee, United States of America; 5 Joint Institute for Neutron Sciences, Oak Ridge National Laboratory, Oak Ridge, Tennessee, United States of America; Institut Curie, France

## Abstract

Rafts, or functional domains, are transient nano- or mesoscopic structures in the exoplasmic leaflet of the plasma membrane, and are thought to be essential for many cellular processes. Using neutron diffraction and computer modelling, we present evidence for the existence of highly ordered lipid domains in the cholesterol-rich (32.5 mol%) liquid-ordered (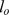) phase of dipalmitoylphosphatidylcholine membranes. The liquid ordered phase in one-component lipid membranes has previously been thought to be a homogeneous phase. The presence of highly ordered lipid domains embedded in a disordered lipid matrix implies non-uniform distribution of cholesterol between the two phases. The experimental results are in excellent agreement with recent computer simulations of DPPC/cholesterol complexes [Meinhardt, Vink and Schmid (2013). Proc Natl Acad Sci USA 110(12): 4476–4481], which reported the existence of nanometer size 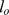 domains in a liquid disordered lipid environment.

## Introduction

**Figure 1:**
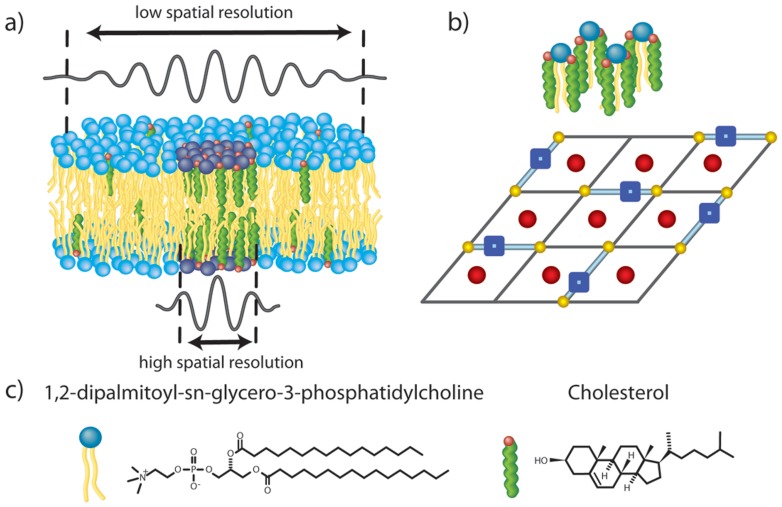
Schematics of the studied systems. a) Schematic of a lipid bilayer containing a lipid domain as studied by neutron scattering techniques using a low (top) and high (bottom) spatial resolution setup. b) In-plane representation of saturated hydrocarbon-chain lipid-cholesterol interactions in accordance with the umbrella model, whereby each lipid is associated with 2 cholesterol molecules. This structural arrangement results when the cholesterol content is 66 mol%. The blue squares represent lipid head groups, the yellow circles correspond to lipid tails, and the red circle are cholesterol molecules. c) Schematic molecular structures of DPPC and cholesterol molecules.

It is widely believed that in the plasma membrane sphingolipids and cholesterol molecules assemble into functional domains, or so-called *rafts* (Figure 1 a) and b)). They are thought to take part in membrane-associated events such as signal transduction, cell adhesion, signalling, cell trafficking and lipid/protein sorting [1]–[13].

While the size, shape, and even the existence of liquid-ordered domains in cell membranes is still a subject of much debate, it is generally agreed that domains coalesce upon cross-linking to form signalling and sorting platforms [14]. Anomalies in raft composition have been associated with various diseases, including atherosclerosis, muscular dystrophy and neurodegenerative disorders, such as Alzheimer’s. Specific proteins associated with disease, such as the amyloid beta (A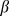) [15] and prions protein [16], as well as various signalling proteins [17]–[19], have been shown to accumulate in rafts. Recently, the membrane’s physical properties have also been suggested to play an important role in modulating protein-protein interactions in rafts [20]–[24].

Cholesterol (Figure 1 c)) is an essential component of eukaryotic cell membranes, and is capable of modulating the membrane’s permeability [25]. In association with rafts, cholesterol has been implicated in cell signalling processes 1,26–29. The phase separation of cholesterol and other biomolecules (e.g., sphingolipids, phospholipids, proteins) into domains is a key element with regard to raft formation in biological systems.

Experimental observations of membrane heterogeneities have proven challenging, as they are thought to be both small and short-lived [4], [30]–[32]. Therefore, in order for experimental techniques to unambiguously observe such structures, they must be capable of simultaneously accessing small (nanometer to micrometer) length scales and fast (nano to microsecond) time regimes. To date, the detection of functional domains in living cells has relied on indirect methods, such as detergent extraction and cholesterol depletion, as they are now believed to be too small to be observed with the presently available microscopy techniques [33].

In this paper we report on neutron scattering and computer modelling experiments that show the existence of nanoscopic domains in the liquid-ordered (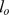) phase of dipalmitoylphosphatidylcholine (DPPC) membranes. This newly developed neutron scattering technique has recently been used to observe co-existing transient nanometer sized domains in a single-component phospholipid membrane at temperatures close to its main phase transition [34].

## Results

### Neutron Experiment

**Figure 2:**
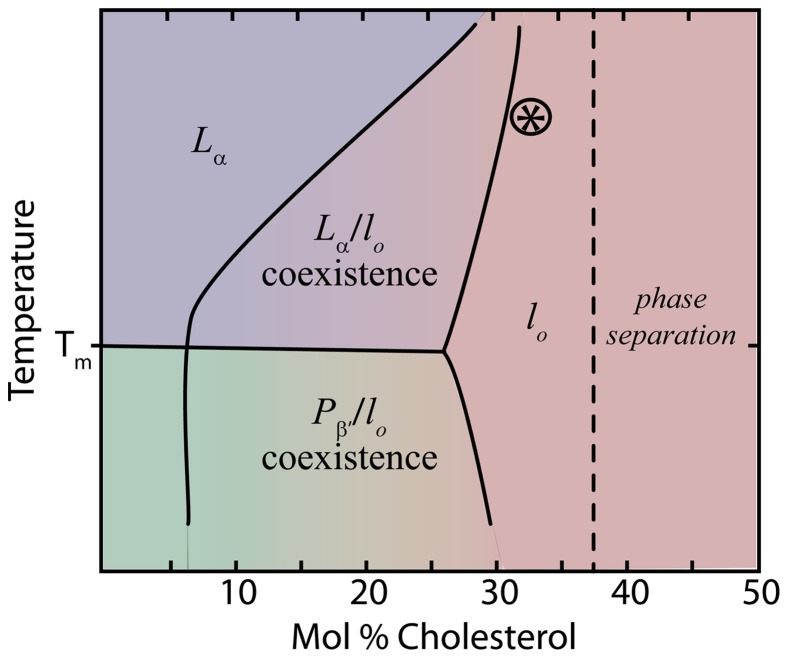
Phase diagram of phospholipid/cholesterol complexes, such as DMPC/cholesterol and DPPC/cholesterol, as reported by, for example, [35]–[40]. Besides the well known gel and fluid phases, the so-called liquid-order phase is observed at high cholesterol concentrations. The 32.5 mol% sample, as depicted by the ⊛, was determined to be in the *l_o_* phase.

To determine the lateral molecular structure, in-plane neutron diffraction was used to measure the liquid-ordered phase of DPPC bilayers at 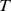 = 50°C and a D_2_O relative humidity of ∼100%, ensuring full hydration of the membranes. Highly oriented, solid supported DPPC membranes containing 32.5 mol% cholesterol were used in this study. A schematic phase diagram of a phospholipid/cholesterol system is shown in Figure 2. Although much is known about lipid-cholesterol mixtures, there is much activity in this area of study due to cholesterol’s role in domain formation [35]–[40]. It has been speculated that the stiff cholesterol molecules align parallel to the hydrocarbon lipid tails and suppress lipid tail fluctuations [41], in turn affecting the membrane’s dynamical properties. Most lipid/cholesterol studies agree that, depending on temperature and cholesterol concentration, three phases are observed, namely: 1) the rigid gel 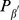 (ripple) phase [42]; 2) the fluid 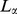 phase; and 3) the liquid-ordered 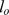 phase. The gel and fluid phases are well known from single component phospholipid bilayers. The 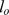 phase is only observed at high concentrations of cholesterol. This phase is somewhat peculiar as it appears to be well ordered (similar to the gel phase), however, the lipids exhibit a diffusion coefficient that is similar to that measured in fluid bilayers.

At low cholesterol content, the bilayers undergo a phase transition from the gel to the fluid phase, as is observed in pure lipid bilayers, and the temperature of the main transition, 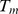, is slightly shifted towards higher temperatures. However, at high cholesterol concentrations (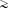
30 mol%), the main transition of liquid-ordered membranes is suppressed, while at intermediate cholesterol concentrations (i.e., between about 10–30 mol%), most studies report a coexistence between gel and 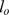, or 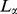 and 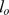 phases. The location on the phase diagram of the sample composition used in the present study is shown in Figure 2. Because the phase boundaries of the phase diagram are not well known, additional experiments were conducted to verify the exact phase of the membranes. The corresponding data is included in the Supporting Information: Specific heat capacity measurements are shown in Figure A in File S1; X-ray reflectivity of a DPPC/37.5 mol% sample is shown in Figure B in File S1; Neutron reflectivity is shown in Figure C in File S1.

Two different neutron scattering setups, were used to study the membranes, namely: (1) a conventional, high energy and momentum resolution setup (i.e. small 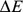 and 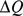); and (2) a low energy and momentum resolution setup (i.e. large 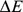 and 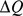), with a high spatial resolution capable of detecting small structures and weak signals. The two setups could be readily switched by changing the incoming neutron wavelength, 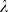, thus eliminating the need for re-aligning the sample. The experiment was predominantly sensitive to the molecular structure and arrangement of the lipid acyl chains, and their contribution to the scattering signal was enhanced by using the chain perdeuterated lipid DPPC-d62. The sample was aligned in the neutron beam such that the scattering vector, 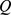, was always in the plane of the membranes. This in-plane component of the scattering vector is referred to as 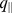.

**Figure 3:**
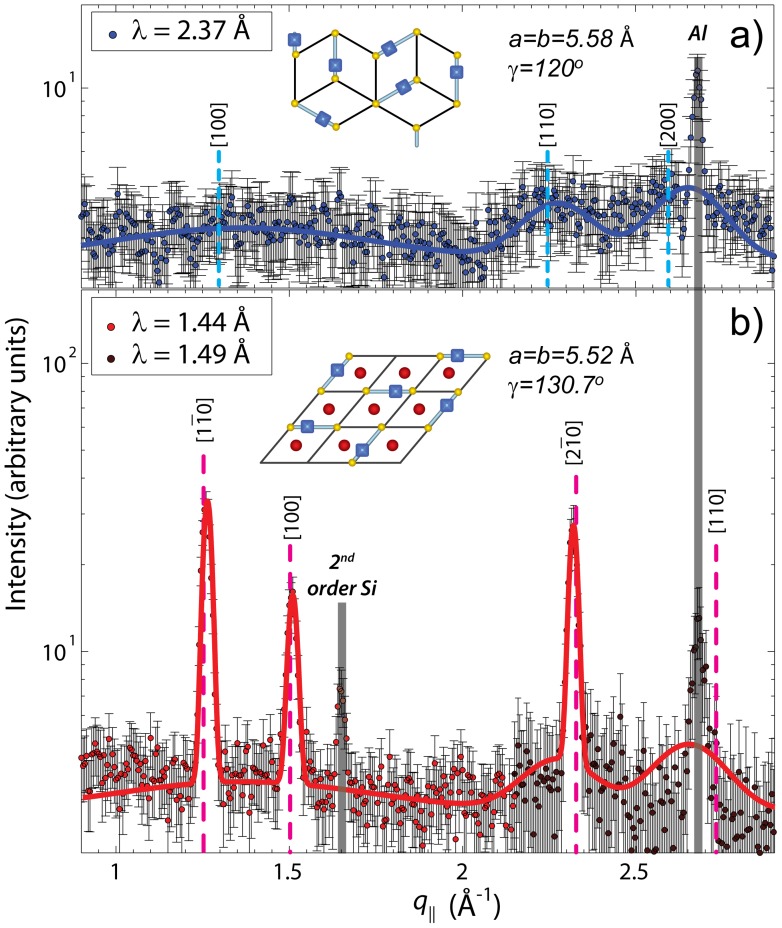
Comparison between the in-plane scans of DPPC-d62 bilayers with 32.5 mol% cholesterol using a) the conventional high energy resolution (small Δ*E*) setup and b) the low energy resolution (large Δ*E*) setup. The data are denoted by circles with the fit shown as a solid line. A disordered structure was observed in a), while the sharp features in b) are indicative of the presence of highly ordered lipid domains. A top view of the corresponding molecular structures are shown in the insets to the Figure using the same symbols as in Figure 1 b); the quasi-Bragg reflections are indicated by vertical dashed lines and their associated Miller indices, [*hkl*]. Peaks resulting from the silicon substrates and the aluminum sample chamber (as described in the Materials and Methods Section) are highlighted in grey, but not accounted for in the fit.

Data taken using the conventional setup are shown in Figure 3 a). The data show a diffraction pattern typical of fluid single and multi-component lipid bilayers [43]–[52]. The broad correlation peaks are signatures of a disordered, fluid structure, with three broad peaks, observed at 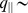
1.36 Å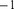,2.28 Å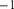, 2.65 Å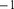, corresponding to the packing of the lipid acyl chains in the membrane’s hydrophobic core. Peaks were fitted using Gaussian profiles and could be indexed by a planar hexagonal unit cell with parameters 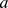 = 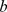 = 5.58 Å and 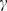 = 120°. Fitting was done using the Powdercell software package [53], [54]. The area per lipid molecule was calculated from the unit cell parameters to be 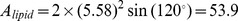 Å^2^ (one unit contains 1 lipid tail and 1/2 headgroup, so the area is doubled in order to represent one lipid molecule). This area per lipid is significantly smaller than the 63.1 Å^2^ [55] and 64.2 Å^2^ [56] found in liquid crystalline DPPC bilayers at 50°C. The cartoon in Figure 3 a) depicts the unit cell and a possible arrangement of the lipid molecules. While the lipid tails were found to arrange on a hexagonal lattice, no long range order was observed for the lipid head groups, unlike the case if DPPC bilayers in the sub gel phase where the lipid headgroups form a two-dimensional lattice [57]–[59].

The diffraction pattern obtained using the low energy resolution (large 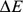) setup is shown in Figure 3 b). In addition to the broad disordered components, three pronounced narrow correlation peaks are visible, indicating the presence of a co-existing, well ordered structure. The positions of the peaks (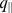 = 1.26, 1.51, and 2.32 Å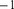) are best described by a monoclinic unit cell of parameters 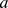 = 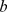 = 5.52 Å and 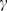 = 130.7°. The cartoon depicts a possible molecular structure. The monoclinic unit cell allows for each lipid to be associated with two cholesterol molecules, in accordance to the umbrella model (discussed below).

The areas determined from the two experimental setups can be compared to a model by Edholm and Nagle [60]. The total area per membrane molecule for a DPPC/cholesterol can be written as the sum of the partial areas of lipid and cholesterol molecules:
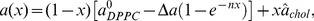
(1)where 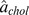 ( = 27 Å^2^) is cholesterol’s area in the asymptotic limit of high cholesterol concentrations, 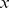, and 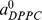 ( = 64 Å^2^) is the area for DPPC at 0 mol% cholesterol. 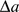 (14 Å^2^) is the change in DPPC area when in contact with cholesterol and 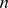 ( = 7.5) is the maximum number of DPPC molecules that can be condensed by a single cholesterol molecule (values were taken from Ref. [60]).

**Figure 4:**
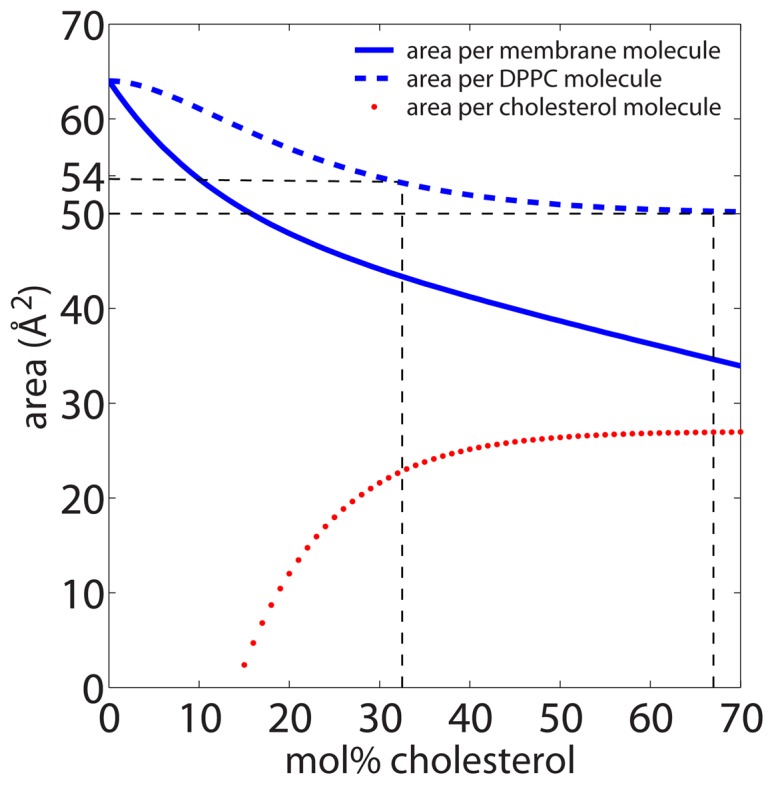
Area per molecule and partial areas for DPPC and cholesterol molecules as function of cholesterol concentration. Curves were calculated using Eq. (1) from [60].

The partial areas of cholesterol and lipid molecules were determined to be 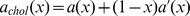 and 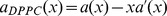; the result of which is plotted in Figure 4. Deuterium labelling was used in our experiment to highlight the structure of the lipid tails. The areas determined in Figure 3 are, therefore, partial lipid areas. The area of 54 Å^2^ for the disordered structure in Figure 3 a) is in excellent agreement with the area per DPPC molecule in Figure 4 in the presence of 32.5 mol% cholesterol. The area per lipid molecule in the the highly ordered domains in Figure 3 b) is calculated to be 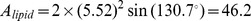 Å^2^. This lipid area is consistent with high concentrations of cholesterol, where lipid condensation reaches a maximum, and is in-line with the umbrella model. We note that this area is also close to the area per lipid published by Tristram-Nagle *et al.* [61], for gel phase DMPC membranes (∼47 Å^2^). The lipids in the highly ordered patches can thus be speculated to have a gel-like structure, with the acyl tails in an all-trans configuration, as will be discussed below.

The equilibrated lipid area is governed by a balance of forces resulting from the headgroup and hydrocarbon chains. In the infinitely long chain length regime, where lipid chain-chain van der Waals attractive interactions dominate (i.e., headgroup electrostatic interactions are negligible), the headgroup has a minimum area due to the steric interactions between the interfacial glycerol-carbonyl groups. However, the observed minimal area is still larger than the optimum packing for all-trans chains of about 40 Å^2^ [62], indicating that the overall lipid area is determined by the headgroup steric limit [63].

The presence of broad correlation peaks in the small 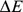 setup and the appearance of additional, sharp reflections in the large 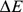 setup are the signature of small, highly ordered lipid domains embedded in a disordered matrix.

### Computer Modelling

The primary reason that small, nanometer sized domains in the 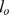 phase have not been previously reported by scattering techniques may be related to the fact that the X-ray and neutron probes coherently average over a given area or volume. Small structures are thus not visible because only coherent spatial averages are observed. A computer simulation can easily model the high and low coherence length setups by calculating the in-plane structure factor, 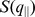, over different areas and comparing the resulting patterns to the experimental diffraction patters in Figure 3.

**Figure 5:**
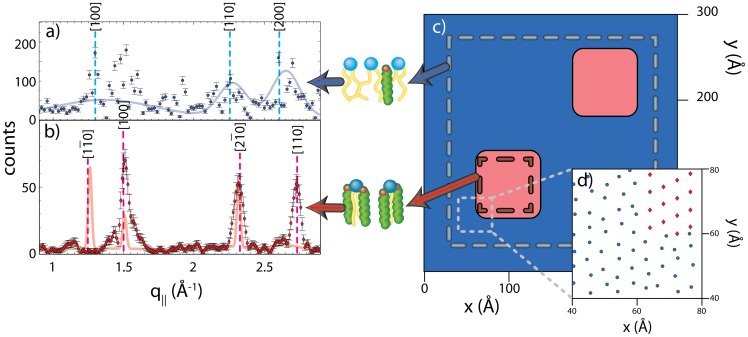
Results of the computer modelling. a) The scattering function *S*(*q||*) integrated over an area of 250*×*250 Å^2^, resulting in a diffraction pattern indicative of fluid, disordered bilayers. b) *S*(*q||*) for an ordered lipid domain of size 70*×*70 Å^2^. Three sharp correlation peaks are observed. The dots represent data obtained from the computer model, while the solid lines correspond to the fits obtained from the neutron scattering experiment in Figure 3 a) and b). Areas of integration are indicated by the blue and red rectangles shown in c). c) Snapshot of a typical configuration in the computer model. The blue region corresponds to the disordered lipid matrix, and the red regions to ordered lipid domains. The total system size was 300*×*300 Å^2^ and contained *∼*2000 lipid molecules, or *∼*4000 lipid tails. The ordered domains were *∼*70*×*70 Å^2^ in size and included *∼*110 lipid molecules. d) A close up view of the computer model system, where the blue circles represent lipid tails in the fluid disordered state and the red diamonds are tails in the *l_o_* phase.

A snapshot of a typical system used for the calculations is schematically shown in Figure 5 c): It consists of a disordered lipid matrix with 2 highly ordered lipid domains. The positions of the lipid acyl chains are represented by dots, as shown by Figure 5 d). The lattice parameters of the disordered and ordered phases were taken from the experiment. Positional disorder was added by introducing a random displacement of the tails from their nominal position: a large amplitude of this random displacement mimics a highly disordered structure, while small or no displacements resulted in highly ordered structures. The aim of the computer model was not to produce an independent model of the structure of the 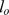 phase, but to demonstrate the effect that coherent averaging over different areas has on the observed scattering.

The high and low energy resolution setups in the neutron experiment were simulated by calculating the scattering function, 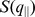, over different simulation areas (Figure 5 c)). The large integration area corresponds to the high energy resolution setup (small 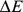). The low energy resolution setup (large 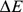), with a higher spatial resolution, on the other hand, was simulated by integrating over an area corresponding to a single domain.

The diffraction pattern in Figure 5 a) shows the result of integrating over an area of 250×250 Å^2^ (containing ∼1350 lipid molecules), as indicated by the blue rectangle in Figure 5 c). The blue area is comprised of the lipid matrix and the embedded domains, resulting in the observation of broad correlation peaks. The obtained scattering pattern, characteristic of a disordered system, is the *coherent* average result, which is comprised of the fluid matrix and the ordered domains.

Figure 5 b) shows the integration of an area corresponding to a single ordered domain (70×70 Å^2^ which includes ∼110 lipid molecules), as indicated by the red rectangle in Figure 5 c). The fit of the experimental diffraction pattern is also shown in b); the calculated peaks positions from Powdercell are marked by the vertical dashed lines. While the computer model well reproduces the positions of the correlation peaks, i.e., the unit cell structure (see Table A in File S1 and Figure D in File S1 for a detailed comparison), the model is too simple to also reproduce the corresponding peak intensities (the form factors). The [1
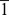
0] was found to be systematically extinct in the computer model while the [110] produced a strong peak, not clearly observed in the experimental data. The most important result was that in both, experiment and computer model, pronounced correlation peaks were observed at a small coherence length indicating the existence of well ordered lipid structures.

## Discussion

The molecular structure of the 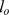 phase in a DPPC membrane containing 32.5 mol% cholesterol was studied using different spatial resolution neutron scattering techniques. Computer modelling revealed that the observed scattering patterns are consistent with a heterogeneous structure composed of ordered nanometer sized lipid domains residing within a disordered liquid matrix.

Initially, the lateral membrane structure was studied by operating the neutron diffractometer in the commonly used low spatial resolution mode, where 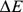 and 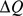 are small (Figure 3 a)). In these experiments neutrons with a long coherence length are used, spatially averaging all in-plane features of the membrane [34].

The scattering pattern from the low spatial resolution setup was indicative of lipids and cholesterol being in a uniform, fluid-like state, as has been previously reported [43]–[47], . The absence of any sharp features in the diffraction pattern indicated that the cholesterol is uniformly distributed in the lipid matrix, with the lipid tails arranging themselves in a hexagonally packed structure on a lattice with parameters 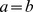 = 5.58 Å and an area per lipid of 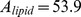 Å^2^. As mentioned, this area per lipid is significantly smaller than that observed in pure DPPC bilayers at 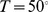
C (63.1 Å^2^ [55] and 64.2 Å^2^ [56]), and is a result of cholesterol’s condensation effect (i.e., ordering of a lipid’s hydrocarbon chains) [38], [39]. In fact, this area per lipid is in excellent agreement with the partial lipid area determined by the model proposed by Edholm and Nagle [60], which is plotted in Figure 4.

In the case of the high spatial resolution setup, however, distinct sharp peaks were observed in both experiment (Figure 3 b)) and computer model (Figure 5 b)). In the case of the experiment, the integration area was determined by the instrumental 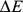 and 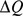, and the corresponding neutron coherence length, 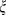. In the simulation, the size of the coherently added area was defined by the length of the distance vector 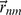 between two lipid tails in the summation of the structure factor 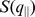. Importantly, both techniques detect the presence of ordered domains embedded in a disordered matrix. In this case, the lipid tails were found to form a monoclinic structure in these domains with lattice parameters 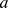 = 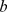 = 5.52 Å and 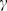 = 130.7°, and a lipid area of 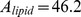 Å^2^.

Several models describing the interaction between lipids and cholesterol are currently being discussed. Of these, the most prevalent are: the umbrella; the complex; and the superlattice model [39], [64]. In the umbrella model [65], [66], each lipid head group can “host” two cholesterol molecules, thus shielding the mostly hydrophobic cholesterol molecule from the aqueous environment. Based on the model for partial areas of lipid and cholesterol molecules by Edholm and Nagle [60], (Figure 4), the partial lipid area decreases significantly towards higher cholesterol concentrations. At the maximum theoretical solubility of 66 mol% cholesterol [65], [68], a partial lipid area of ∼50 Å^2^ is calculated, comparable to the ∼47 Å^2^ lipid area of DPPC in gel phase membranes [61]. M’Baye *et al.* addressed this point [69] and concluded that cholesterol, due to its specific H-bonding interactions with lipids and its ability to fill voids in lipid bilayers, may efficiently expel water molecules from the highly ordered gel phase to form the 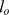 phase. As early as 1987, Hjort Ipsen *et al.* [70] reported that the bilayer at high cholesterol concentrations behaves as a liquid with greatly reduced membrane-area compressibility. NMR measurements have shown that the deuterium order parameters approach a value of 0.5, typical of an all-trans rotating hydrocarbon chain. The hydrocarbon chain organization determined from the correlation peaks observed by experiment in Figure 3 b) is consistent with an ordered structure containing elevated amounts of cholesterol, where all the voids in the monoclinic lipid lattice are filled with cholesterol molecules, as depicted in the cartoons to Figures 1 b) and 3 b). This suggests that the lipid domains are comprised of lipids with highly ordered hydrocarbon chains.

Recent observations have suggested that in the mammalian plasma membrane, small domains residing within ordered lipid phases are in dynamic equilibrium with the less ordered portions of the membrane [8]. Armstrong *et al.* studied the nanoscale dynamics of the lipids making up domains [52]. They reported that on the nanometer length scale, cholesterol rich domains seem to be softer than fluid phase bilayers, and at the same time, are better ordered than lipids in the gel phase. The present data taken together with the study by Armstrong *et al.* [52], add significant weight to the recently proposed plasma membrane model.

We note that it is not straightforward to directly relate the coherence length of the neutron beam to domain size. The coherence length of a neutron beam was first measured by Kaiser, Werner and George [71] by neutron interferometry. In one dimension (1D), a neutron in the neutron beam can be described by a 1D wave package, 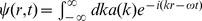. If the amplitude, 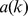, is approximated by a Gaussian amplitude function, 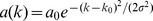, the wave package will have a Gaussian shape [71]. In this simple model, the coherence length, 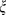, can be thought of as the FWHM of the Gaussian wave package, FWHM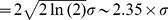. The intensity measured at the detector is the result of the superposition of the different scattered waves; for two waves: 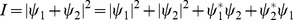. The first two terms describe incoherent scattering. Coherent, Bragg scattering is the result of the cross terms in the equation. If there is no overlap between the wave function, only incoherent scattering takes place. The two wave functions will certainly overlap if they are closer together than the FWHM, which corresponds to the coherence length. However, the total spatial extent of the wave function is larger than the FWHM.

Additionally, the wave functions are commonly approximated by Gaussian functions as these are limited in space, and their Fourier Transform can easily be calculated. In a more realistic model, the wave package may fall off much more slowly than a Gaussian package, which would drastically increase the spatial extent of the package, making the cross terms observable at distances much larger than the coherence length (the FWHM) of the wave package.

The Scherrer equation [72] is often used to determine particle sizes in diffraction experiments. However, it is well known (see for instance [73]) that this equation can only be applied for average sizes up to about 1000–2000 Å, when the broadening becomes comparable to the experimental resolution. We note that this critical length scale is comparable to the coherence length in a typical X-ray diffraction experiment, as listed in Table I. The reason is that the wavelength and wavelength resolution are important parameters for the instrumental peak broadening, as well as the coherent properties of the X-ray or neutron beam. Scherrer’s equation is known to fail when the coherence length becomes comparable to the particle size as the measured peak widths become resolution limited in the experiment. This effect was observed in the peaks in Figure 3 b) (and also in the previous experiment by Armstrong *et al.* [34]).

It is non-trivial and, at this point, not possible to quantitatively relate the domain size to the coherence length of the neutron beam. The full width at tenth of maximum of a Gaussian (FWTM), FWTM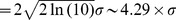, contains 90% of the Gaussian area and may be used as an estimate of the domain size. The experimental neutron coherence length of ∼35 Å would then give a tentative domain size of ∼65 Å. As a result, domain sizes of ∼70 Å were used in the computer simulations in order to compare to experiment.

Our results can be compared to a recent computer simulation study by Meinhardt, Vink and Schmid [74] of a DPPC/cholesterol system. By using a coarse grained model that included 20,000 lipid molecules, a microemulsion-type state was observed that contained nanometer-sized 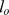 domains in a liquid disordered environment. The liquid ordered phase in lipid membranes was previously modelled as a homogenous phase. The computational work has two important results. Firstly, raft formation was observed in a binary system, while it was previously thought that raft-mixtures required the presence of several different types of lipids and cholesterol. Secondly, small, nanometer sized domains on the order of 100 Å were observed. These findings are in excellent agreement to the experimental results presented here. In future experiments the size of the domains will be determined from measurements of the corresponding structure factor using small angle neutron or X-ray scattering experiments.

### Conclusions

By combining neutron scattering and computer modelling we present evidence suggesting that the liquid-ordered, 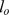, phase in DPPC membranes contains highly ordered lipid domains. DPPC bilayers with 32.5 mol% cholesterol were prepared in their high cholesterol 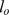 phase, but below the critical concentration at which phase separation takes place. Experiments and simulations were performed using two different spatial resolutions. A small 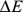 and 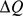 neutron setup, which integrated over large areas of membrane, gave rise to a diffraction pattern typical of fluid-like, disordered systems. Similar data were reported previously from neutron and X-ray scattering experiments, and suggest a homogeneous distribution of cholesterol molecules within the lipid matrix.

Increasing the spatial resolution of the experiment by using a configuration with a large 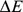 and 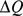 resulted in a drastic decrease of the coherence length of the neutron beam, enabling smaller membrane patches to be studied. This setup resulted in distinct correlation peaks due to a local ordering of the lipid acyl chains. The reflections could be modelled using a simulated system of small, ordered nanoscopic lipid domains. We speculate that these domains are saturated with cholesterol molecules, i.e., two cholesterol molecules per lipid molecule, as suggested by the umbrella model. The elastic neutron scattering experiments taken in this study, together with recent inelastic experiments [52] suggest that small domains in ordered lipid phases are in dynamic equilibrium with the less ordered parts of the membrane.

## Materials and Methods

### Sample Preparation

Chain perdeuterated 1,2-dipalmitoyl-sn-glycero-3-phosphocholine (DPPC-d62) was used to enhance the intensity of the coherent out-of-plane and in-plane neutron Bragg diffraction peaks. Highly oriented multi-lamellar stacks of DPPC with 32.5 mol% cholesterol were prepared on 2″ single-side polished Si wafers of thickness 300 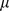
m. A solution of 16.7 mg/mL DPPC-d62 with 32.5 mol% cholesterol in 1∶1 chloroform and 2,2,2-trifluoroethanol (TFE) was prepared. The Si wafers were cleaned by alternate 12 minute sonications in ultra pure water and methanol at 313 K. This process was repeated twice. The cleaned wafers were placed on a heated sample preparation surface, which was kept at 50°C. This temperature is well above the main phase transition for DPPC, thus the heated substrates ensured that the lipids were in the fluid phase after deposition. 1.2 mL of the lipid solution was deposited on each Si wafer and allowed to dry. The wafers were kept under vacuum overnight to remove all traces of the solvent. Samples were then hydrated with heavy water, D_2_O, and annealed in an incubator at 328 K for 24 hours. Following this protocol, each wafer contained ∼3000 highly oriented stacked membranes with a total thickness of ∼10 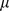
m. Sixteen such Si wafers were stacked with 0.6 mm aluminum spacers placed in between each wafer to allow for the membranes to be properly hydrated. The “sandwich” sample was kept in a sealed temperature and humidity controlled aluminum chamber. Hydration of lipid membranes from water vapour was achieved by independently adjusting the temperature of the heavy water reservoir, the sample and the chamber cover. Temperature and humidity sensors were installed close to the sample. A water bath was used to control the temperature of the different water reservoirs, and the temperatures of the sample and its cover were controlled using Peltier elements. The hydration of the sample was estimated from the lamellar spacing 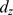 to better than 99.6%.

**Figure 6:**
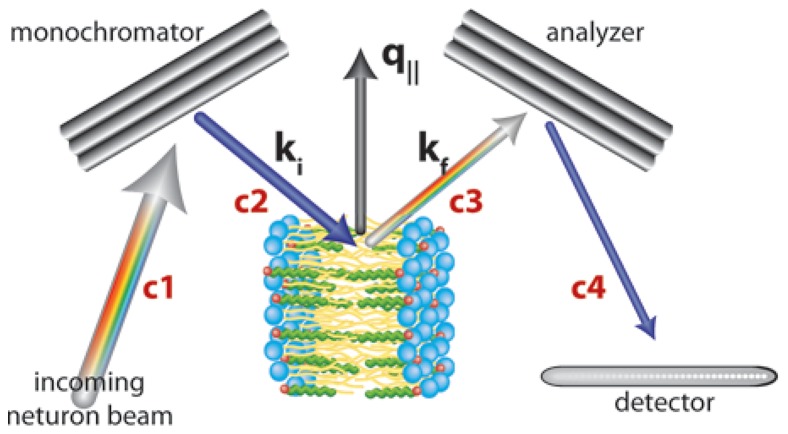
Geometry of the triple axis spectrometer. Orientation of the sample for in-plane scans, such that the scattering vector, *Q*, lies in the plane of the membrane (*q_||_*). *k_i_* and *k_f_* are the incident and final neutron wave vectors (*k = *2*π = λ*) and the c’s denote the location of collimators along the beam line.

The samples were mounted vertically in the neutron beam such that the scattering vector, *Q*, could either be placed in the plane of the membrane (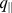), or perpendicular to it (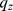). The out-of-plane and in-plane structures could be measured by simply rotating the sample by 90 degrees, as shown in Figure 6.

The main transition temperature, 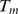, of DPPC-d62 was reported to occur at 310.5 K, [75] a value slightly lower than its protonated counterpart (T = 314.4 K) [75], [76]. The temperature of the pre-transition from the 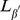 to the 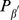 phase in deuterated DPPC-d62 was determined to be 302.9 K [75]. All measurements reported here were done at T = 323.2 K (50°C), well above 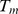.

#### Neutron experiment

Experiments were conducted using the N5 triple-axis spectrometer at the Canadian Neutron Beam Centre (Chalk River, ON, Canada). The three axes of the spectrometer refer to the axis of rotation of the monochromator, the sample and the analyzer. The incident and final neutron energies are defined by the Bragg reflections from pyrolytic graphite (PG) crystals. The divergence of the neutron beam was controlled by Soller collimators. A schematic of the instrument’s configuration is shown in Figure 6. The instrumental parameters for the two setups used in this experiment are listed in Table I.

The 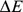 and 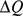 of a neutron triple-axis spectrometer are determined by: (1) the incident energy of the neutron beam; (2) the divergence of the neutron beam; and (3) the wavelength resolution of the monochromator and analyzer. Collimation was kept constant during the course of the experiment. Small and large 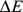 setups were achieved by varying the incident energy of the incoming neutrons. The longitudinal coherence length of the neutron beam, 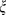, is defined by 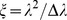 [77]. For a neutron spectrometer with incident neutron energy 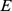 and instrumental energy resolution 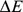, 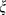 can be estimated to be 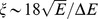 [34], where 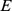 and 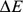 are in meV. The transverse coherence length 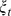 can be estimated to be 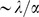, where 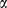 is the divergence of the neutron beam. Long transverse coherence lengths of several micrometers are achieved in small angle neutron scattering (SANS) instruments by pinholes. The transverse coherence length in our setup was of the order of ∼5 Å, and is small compared to the longitudinal coherence. Note, that the reason for the typically low monochromaticity of neutron beams is to avoid further compromising the already low flux “white” neutron beam, a situation that is very different for synchrotron X-rays.

Switching between the high and low energy resolution setups was done by changing instrumental settings of the neutron triple-axis spectrometer, which has an effect on 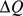 and 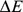 of the beam. A smaller neutron wavelength leads to strongly relaxed 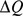 and 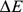. In addition, the longitudinal coherence length of the neutron beam decreases. The most significant changes between the high and low energy resolution setups are: (1) a more efficient integration over larger 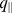 ranges to enhance small signals; and (2) a reduction of the coherently added scattering volume.

Typical values for 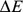, 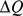, and coherence length for (cold) neutron diffraction and X-ray diffraction experiments are also included in Table 1. Typical X-ray photon energies are between ∼4 and ∼20 keV, with wavelength resolutions of 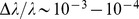, which are achieved by silicon monochromators. Cold neutrons are usually used for experiments in biological materials, with typical energies of 4.2 meV and a monochromatization of ∼1%. While these techniques appear to be favourable to pick up small signals from small domains (because of their large 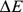 and at the same time, small 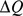, the long coherence length 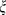 prevents high spatial resolution. Previously, setup ? was successfully used in [34] for the study of lipid nanodomains in membranes.

**Table 1:** Instrumental parameters for the low (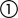) and high (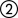) energy resolution setups used. PG(002) crystals were used for both the monochromater and analyzer.

Setup	*λ*	E	Δ*E*	Δ*Q*	*ξ*
	(Å)	(meV)	(meV)	(Å^–1^)	(Å)
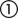	1.44	39.5	3.521	0.034	32.3
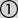	1.49	36.8	3.173	0.032	34.6
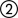	2.37	14.6	0.757	0.020	91.3
Neutron Diffraction	4.2	4.66	∞	0.005	420
X-ray Diffraction	1.54	8.041⋅10^6^	∞	0.001	∼3000

The collimation was set to (c1–c2–c3–c4): 30-18-28-60 (in minutes). Energy and *Q*-resolution (given as FWHM) were calculated using the ResLib software package by A. Zheludev [80] adapted to the N5 spectrometer. For comparison, typical values for energy, 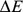, 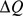, and coherence length for (cold) neutron diffraction and X-ray diffraction experiments are also included.

#### In-plane peak assignments

**Figure 7:**
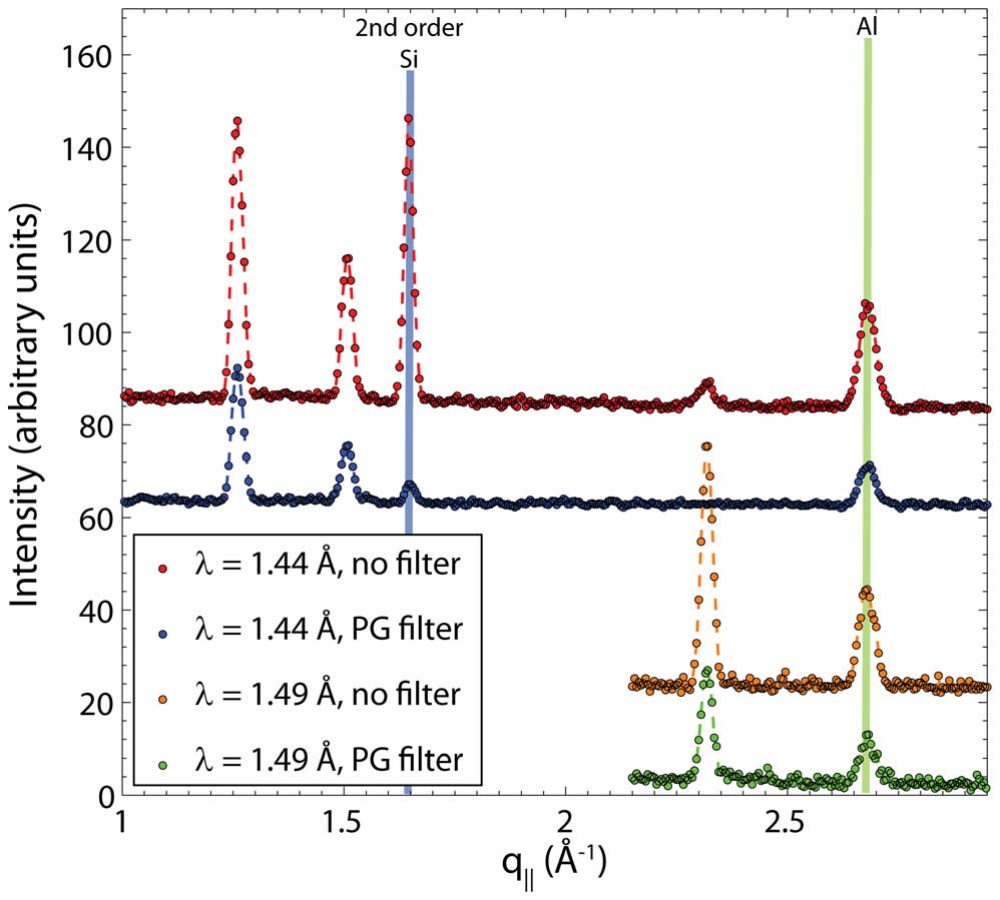
In-plane diffraction data and peak assignments. Data were taken with and without a PG filter, which was used to suppress higher order reflections.

Figure 7 shows in-plane diffraction over an extended range of in-plane momentum transfers, (1 Å
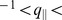
3 Å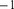), measured at neutron wavelengths of 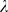 = 1.44 Å and 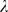 = 1.49 Å. Several Bragg peaks are observed at 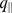-positions of 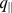 = 1.26 Å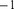, 1.51 Å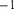, 1.65 Å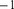, 2.32 Å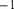 and 2.70 Å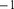. In order to unambiguously assign scattering signals to the membrane system, the origin of the peaks was determined.

The observed Bragg peaks can be the result of: (1) scattering from the sample; (2) scattering from the aluminum sample environment or the silicon substrate; or (3) the result of multiple scattering. The positions of the aluminum and silicon Bragg reflections were calculated based on their known crystal structures. Aluminum forms a face-centred cubic lattice (space group 203) with lattice parameter 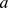 = 4.0497 Å. Silicon forms a cubic lattice (space group 227) with lattice parameter *a* = 5.4309 Å. The positions of the corresponding aluminum and silicon Bragg peaks are shown in Figure 7. The Bragg peak at 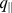 = 2.70 Å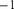 can be assigned to aluminum, most likely from the windows of the humidity chamber and sample holder that were used to house the silicon wafers.

Bragg’s equation 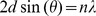 allows for Bragg peaks at integer values of *n*. This is a particularly relevant point when crystal monochromators are used to monochromate the incoming neutron beam, as was the case in our experiment. If the instrument is set up for a wavelength of 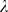 = 2.37 Å, for example, 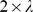 and 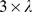 also happen to fulfil Bragg’s law, leading to reflections at approximately one half and one third of the original calculated positions. The reflection at 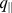 = 1.65 Å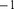 fulfils this condition, agreeing with the position of the silicon [220] reflection peak measured at a wavelength of 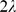. Higher order wavelengths can be suppressed in neutron experiments by using filters that only transmit the original wavelength and strongly absorb all other wavelengths. Figure 7 also includes data measured with a PG filter [78]. While the intensity of all peaks decreased, the peak at 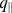 = 1.65 Å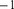 was drastically reduced in the presence of the filter, and was therefore assigned to a second order Bragg reflection from the silicon substrate. We note that the silicon wafers used in this experiment were single crystals. However, due to multiple scattering events [79], Bragg reflections can be observed even when the corresponding crystal axis is not aligned in the scattering plane.

Based on the above analysis, three peaks in Figure 7, namely those at 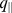 = 1.26 Å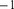, 1.51 Å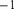 and 2.32 Å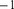, can be unambiguously assigned to scattering from the membrane structure, and were the only ones used to determine the lipid tail structure.

#### Computer modelling

The 2D model started by generating the disordered matrix. The matrix was filled with a hexagonal lattice based on the experimentally obtained lattice parameters *a* = *b* = 5.58 Å and 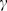 = 120°. The disordered liquid state was then generated by random displacements from the initial lattice positions. The random displacements, 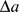, follow a Gaussian distribution with a FWHM of 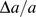 = 8%. The computer calculations were setup in Matlab using Matlab’s normrnd Normal random number generator. 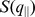 was calculated in 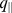 steps of 0.001 Å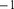. The underlying grid had a resolution of 0.2 Å.

The simulation liquid crystalline domains of different sizes were then generated according to the the experimental results, with lattice parameters *a* = *b* = 5.52 Å and 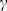 = 130.7°, and randomly placed within the lipid matrix. A typical system size involved an area of 300×300 Å^2^, and included ∼4000 lipid hydrocarbon chains, or equivalently, ∼2000 lipid molecules. A typical domain size was ∼70×70 Å^2^. A sketch of a snapshot of a typical simulated system is shown in Figure 5 b).

Diffraction patterns were determined by calculating the structure factor
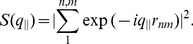
(2)


The length of the largest vector 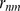 determined the size of the patch. A large 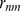 corresponds to the high resolution setup in Figure 3 b), a small 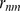 to the low resolution setup. The area of summation was randomly placed on the system and 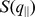 was calculated. The calculation was repeated 25 times and averaged for the final result. The calculations took ∼250 minutes on a quad core desktop computer.

## Supporting Information

File S1 **Electronic Supplementary Material to: The Observation of Highly Ordered Domains in Membranes with Cholesterol.**
(PDF)
