# Supplementary material for: The Observation of Highly Ordered Domains in Membranes with Cholesterol
Source: PLoS One. 2013 Jun 18;8(6):e66162. doi: 10.1371/journal.pone.0066162 (PMC3688844; doi:10.1371/journal.pone.0066162)
Supplement: File S1 — Electronic Supplementary Material to: The Observation of Highly Ordered Domains in Membranes with Cholesterol. (PDF) [file pone.0066162.s001.pdf]

Supporting Information to: The Observation of Highly Ordered Domains in Membranes with Cholesterol

Clare L. Armstrong,[1,*] Drew Marquardt,[2,3] Hannah Dies,[1]
Norbert Kučerka,[3] Zahra Yamani,[3] Thad A. Harroun,[2]
John Katsaras,[4,3,5] An-Chang Shi,[1] and Maikel C. Rheinstädter[1,3,†]

[1] *Department of Physics and Astronomy, McMaster University, Hamilton, Ontario, Canada*

[2] *Department of Physics, Brock University, St. Catherines, Ontario, Canada*

[3] *Canadian Neutron Beam Centre, National Research Council of Canada, Chalk River, Ontario, Canada*

[4] *Neutron Sciences Directorate, Oak Ridge National Laboratory, Oak Ridge, Tennessee, United States of America*

[5] *Joint Institute for Neutron Sciences, Oak Ridge National Laboratory, Oak Ridge, Tennessee, United States of America*

[*] armstc5@mcmaster.ca

[†] rheinstadter@mcmaster.ca

VERIFICATION OF THE LIQUID-ORDERED STATE

Additional measurements were conducted to verify that the DPPC/32.5 mol% cholesterol sample was in the l_o phase. Measurements of specific heat capacity are sensitive to phase transitions. In particular the pre- and main transitions of lipid membranes are easily detected and have distinct signals [1]. Thermograms were collected using a Shimadzu DSC-60 and a TA-60WS Thermal Analyzer. The calorimeter was calibrated using octadecane, which has a melting temperature of 27.95°C. An indium (In) standard was used in the heater power calibration, with a transition enthalpy of 28.6 J/g. Data were collected at a scan rate of 5°C/min and a sampling time of 1 second.

DPPC and cholesterol (at a concentration of 32.5 mol%) were co-dissolved in organic solvent and the solvent was removed by a gentle stream of N_2 followed by several hours under vacuum. The mixture was hydrated with ultra pure water to a DPPC concentration of 0.166 mg/μL. It was then put through 5 freeze/thaw cycles. An aluminum hermetically sealed pan was loaded with 30 μL of the lipid/cholesterol solution. The same procedure was followed for a pure DPPC sample. Background was determined by a scan containing only 30 μL of ultra pure water.

Heat capacity data are shown in Figure A. A pure DPPC membrane was measured as a reference and shows signals consistent with the pre- and main transitions of DPPC bilayers, in excellent agreement with literature values: The main transition temperature, T_m, of DPPC was determined to be T = 314.4 K [2, 3]; the temperature of the pre-transition ($L_{\beta'}$ to the $P_{\beta'}$) was determined to be 303.1 K [3]. The absence of a transition in DPPC bilayers with 32.5 mol% cholesterol is indicative that the cholesterol concentration of 32.5 mol% is high enough to induce l_o phase.

As marked in the schematic phase diagram shown in Figure 2 of the main paper, phase separation has been speculated at cholesterol concentrations of ∼40 mol% and above.

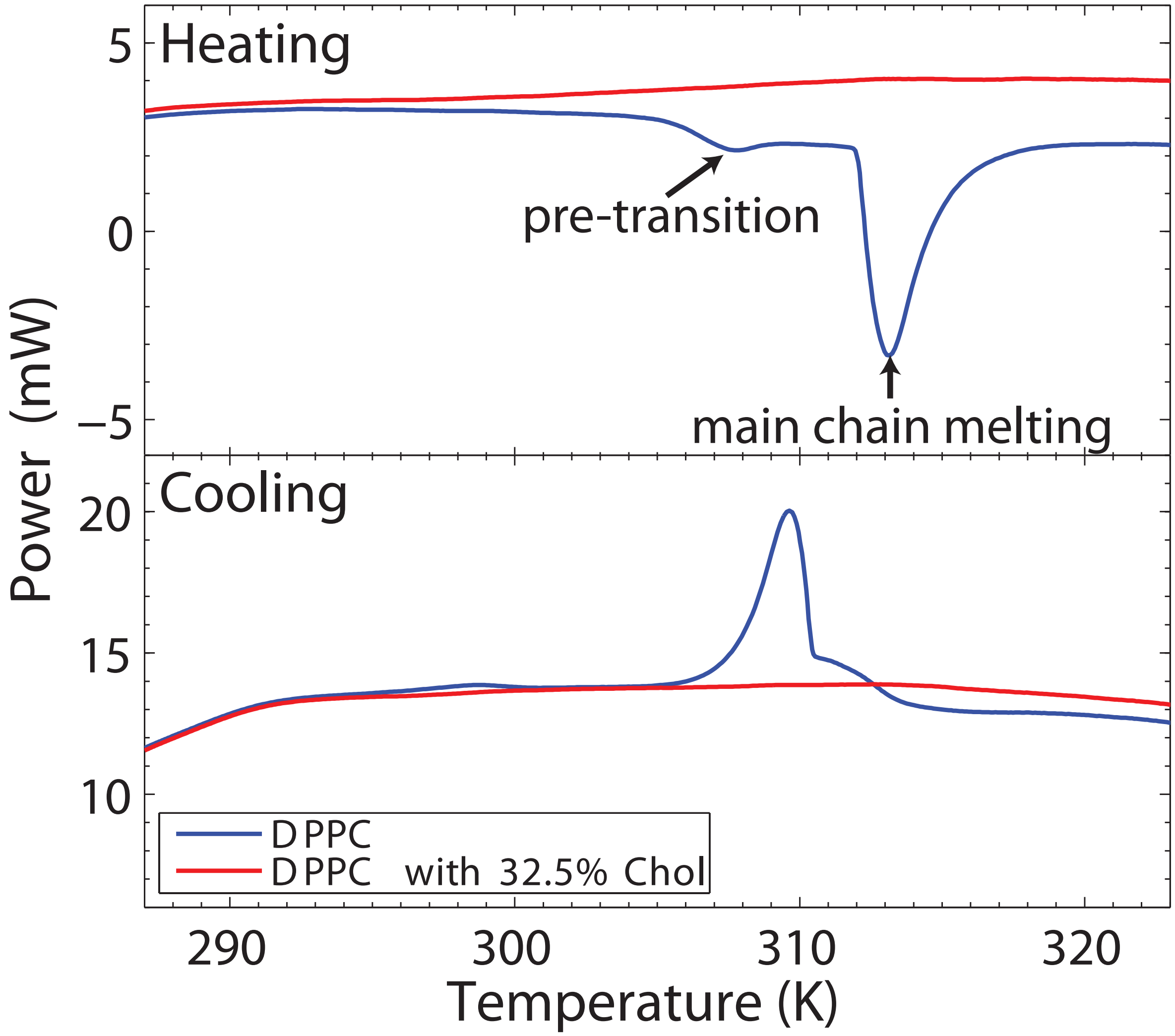

FIG. A. Specific heat capacity data measured upon heating and cooling. A pure DPPC bilayer scan is included for comparison. While the DPPC sample (blue) shows signals corresponding to the pre- and main transition observed in a variety of lipids, no transition was observed in DPPC bilayers containing 32.5 mol% cholesterol (red).

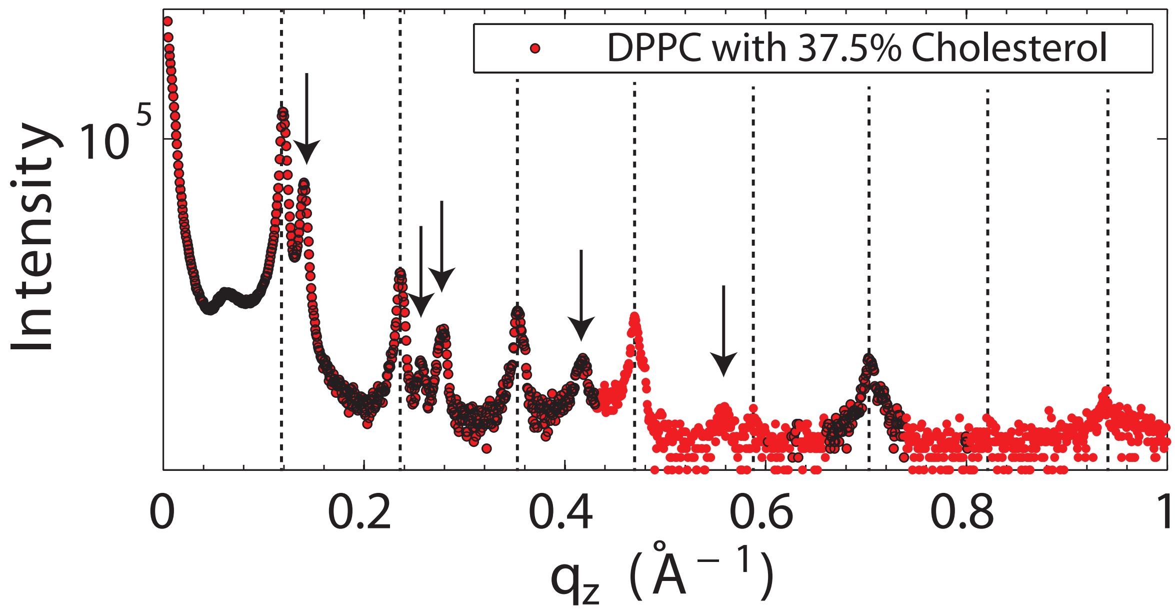

FIG. B. X-ray reflectivity for DPPC with 37.5 mol% cholesterol. Peaks due to the lamellar structure are marked by vertical lines. Additional peaks are observed and marked by arrows, an indication of a phase separated structure.

X-ray reflectivity is an excellent probe for determining resultant structures. X-ray scattering data was obtained using the Biological Large Angle Diffraction Experiment (BLADE) in the Laboratory for Membrane and Protein Dynamics at McMaster University. BLADE uses a 9 kW (45 kV, 200 mA) CuKα rotating anode at a wavelength of 1.5418 Å. Both source and detector are mounted on movable arms, such that the membranes remain horizontal during the measurements. Focusing multi-layer optics yield a high intensity parallel beam with monochromatic X-ray intensities up to 10^{10} counts/(mm$^2\times$s). This beam geometry provides optimal illumination of the solid supported membrane samples, thus maximizing the scattering signal. A reflectivity scan of the 32.5 mol% sample used in this study was taken at 20°C and 50% relative humidity. A number of pronounced Bragg peaks due to the lamellar membrane structure were observed, indicative of a well organized and uniform lamellar morphology.

A sample with a higher cholesterol concentration (37.5 mol%) was prepared for comparison. The X-ray reflectivity curve from this sample is shown in Figure B. Additional peaks are observed at this cholesterol concentration, which are indicative of co-existing, phase separated structures, as was previously discussed [4]. Taking into account this data, it is safe to

assume that the 32.5 mol% cholesterol sample is in the l_o phase, as indicated on the phase diagram (Figure 2).

The quality and structure of the 32.5 mol% cholesterol sample was also checked using out-of-plane neutron reflectivity scans. Reflectivity was measured on N5 in-situ using the same setup as for the in-plane measurements. This was accomplished by simply rotating the sample by 90°. Figure C a) shows reflectivity scans measured before and after sample

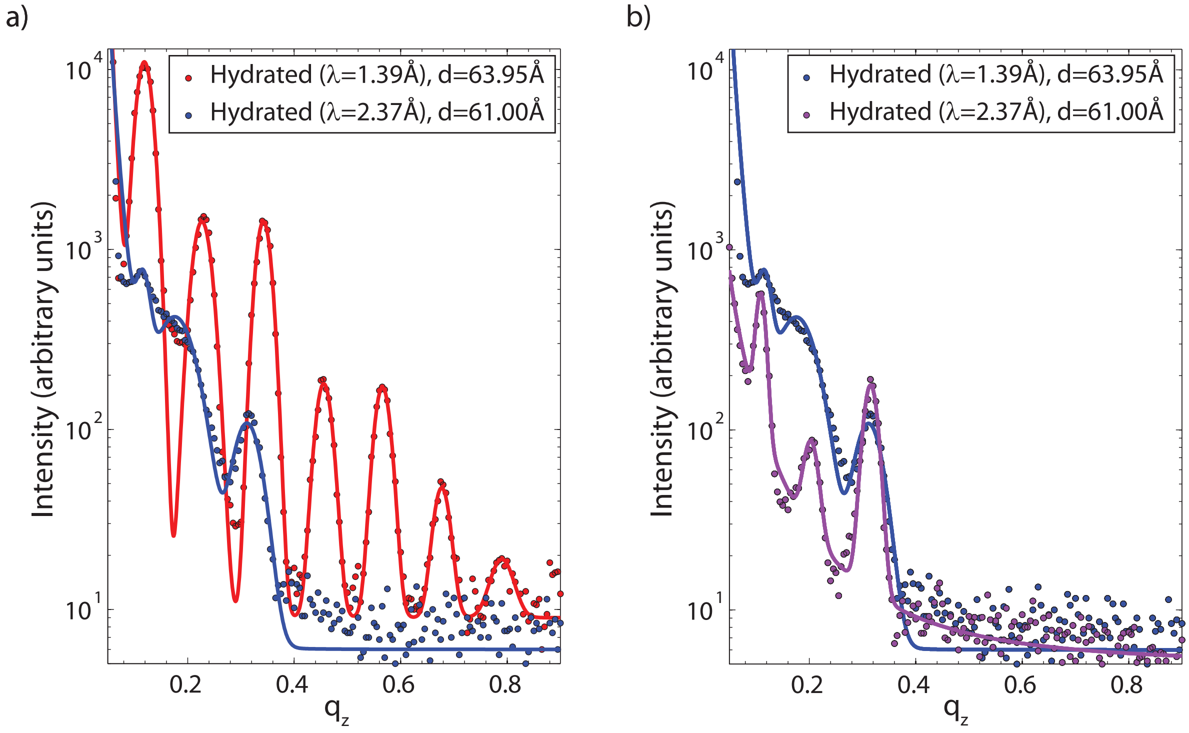

FIG. C. Out-of-plane neutron reflectivity data measured in parallel to the in-plane data. a) A dry and a hydrated DPPC sample with 32.5 mol% cholesterol. b) Reflectivity data from the hydrated sample measured using the high and low resolution setup.

hydration. Data were collected at a temperature of 50°C. Seven orders of lamellar Bragg peaks are observed for bilayers in the dry state, and a d_z-spacing of d_z=56.1 Å can be determined from the symmetric and equally spaced reflections. After hydration, the higher order reflections were found to be strongly suppressed, indicative of the onset of strong bilayer fluctuations induced by the water. A d_z-spacing of d_z=64.0 Å was determined.

Figure C b) depicts neutron reflectivity scans measured using the two different setups (① and ②) used for the in-plane investigations. While the Bragg peaks can be well resolved using the high resolution setup, peaks appear significantly broader when using the low

resolution setup. However, the two scans are in agreement within the different resolutions.

The present results can be compared to a recent study by Gallová *et al.* who used multilamellar DPPC liposomes with 33.3 mol% cholesterol [5]. The lamellar d_z-spacing and phase transitions were investigated using X-ray diffraction. No main transition was observed at this cholesterol concentration, in agreement with our calorimetric measurements. The observed d_z-spacing of 68 Å is slightly larger than the 64 Å that we observed (Figure C). However, Gallová *et al.* mentioned that their d_z values were slightly larger than those reported from measurements of solid supported DPPC bilayers published in the literature.

VALIDATION OF THE STRUCTURE DETERMINED BY COMPUTER MODELLING

The result of the computer model calculations in Figure 5 of the main text can be compared to crystallographic lattice calculations. Calculations were done using the Powdercell software package [6, 7]. Calculated positions of Bragg peaks for the two observed lattices are given in the following Table. The diffraction pattern obtained by simulation is plotted

	disordered matrix			lipid rafts					
$[hkl]$	[100]	[110]	[200]	$[1\bar{1}0]$	[100]	$[2\bar{1}0]$	$[2\bar{2}0]$	[110]	[200]
Neutron Experiment	1.36	2.28	2.65	1.26	1.51	2.32	-	-	-
Computer Modelling	1.46	2.22	2.67	-	1.50	2.32	-	2.73	3.00
Lattice Calculation	1.30	2.25	2.60	1.25	1.50	2.32	2.51	2.73	3.00

TABLE A. Comparison between structures determined by experiment, simulations and lattice calculations. The $q_{||}$-positions of the $[hkl]$ Bragg peaks are given in Å^{-1}. The $[h\bar{h}0]$ reflections, such as the $[1\bar{1}0]$ and the $[2\bar{2}0]$ reflections, are systematically extinct in the computer simulations, as explained in the text.

in Figure D. The vertical lines mark the positions of the calculated Bragg peaks. Simulations and exact lattice calculations show excellent agreement with each other up to the

higher order reflections, proving the correctness of the structure factor calculations. The $[h\bar{h}0]$ reflections, such as the $[1\bar{1}0]$ and the $[2\bar{2}0]$ reflections, are systematically extinct in the computer simulations. The product $q_{||}r_{nm} = (ha^* + kb^*) \cdot (na + mb)$ simplifies to $(n+m)a^2$. The calculated structure factor $S(q_{||})$, therefore has a minimum for $n = -m$, i.e., for reflections with $h = -k$. The $[1\bar{1}0]$ peak was, however, observed in the experimental data shown in Figure 3 of the main paper. While the model well reproduced the structure, i.e., the peak positions, it failed to also reproduce the corresponding peak intensities because the form factor used was too simple.

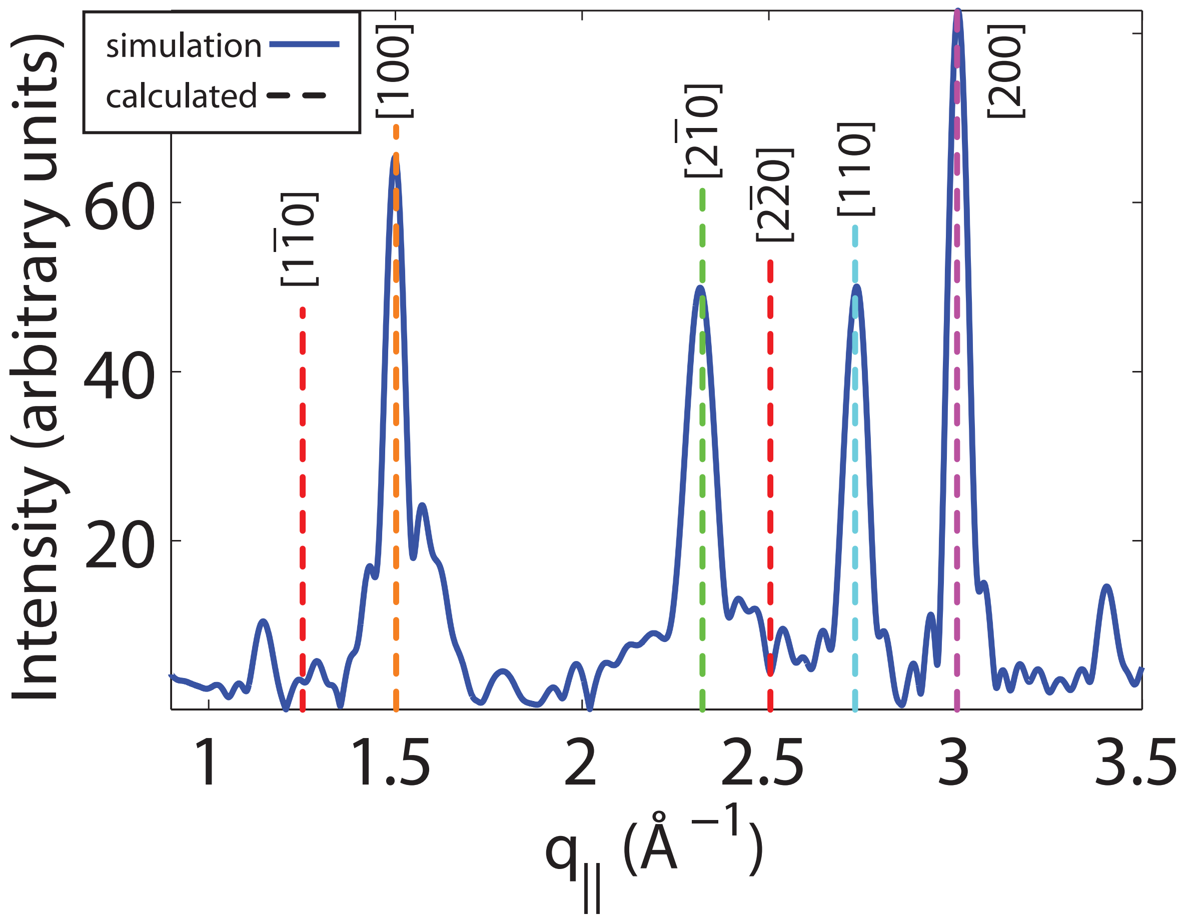

FIG. D. Calculated and simulated diffraction patterns of the monoclinic (a=b=5.52 Å, γ=130.7°) structure. Data show excellent agreement between simulations and crystallographic lattice calculations.

[1] Heimburg T (2007) Thermal biophysics of membranes. Weinheim: Wiley-VCH.

[2] Mabrey S, Sturtevant J (1976) Investigation of phase transitions of lipids and lipid mixtures by high sensitivity differential scanning calorimetry. Proc Natl Acad Sci USA 73: 3862-3866.

[3] Katsaras J, Epand RF, Epand RM (1997) Absence of chiral domains in mixtures of dipalmitoylphosphatidylcholine molecules of opposite chirality. Phys Rev E 55: 3751-3753.

[4] Mills T, Tristram-Nagle S, Heberle F, Morales N, Zhao J, et al. (2008) Liquid-liquid domains in bilayers detected by wide angle x-ray scattering. Biophys J 95: 682-690.

[5] Gallová J, Uhríková D, Kučerka N, Doktorovová S, Funari S, et al. (2011) The effects of cholesterol and β-sitosterol on the structure of saturated diacylphosphatidylcholine bilayers. European Biophysics Journal 40: 153-163.

[6] Kraus W, Nolze G (1996) *POWDER CELL* – a program for the representation and manipulation of crystal structures and calculation of the resulting X-ray powder patterns. Journal of Applied Crystallography 29: 301–303.

[7] (2000). http://www.ccp14.ac.uk/ccp/web-mirrors/powdcell/a_v/v_1/powder/e_cell.html.
